# Androgen receptor signaling promotes Treg suppressive function during allergic airway inflammation

**DOI:** 10.1172/JCI153397

**Published:** 2022-02-15

**Authors:** Vivek D. Gandhi, Jacqueline-Yvonne Cephus, Allison E. Norlander, Nowrin U. Chowdhury, Jian Zhang, Zachary J. Ceneviva, Elie Tannous, Vasiliy V. Polosukhin, Nathan D. Putz, Nancy Wickersham, Amrit Singh, Lorraine B. Ware, Julie A. Bastarache, Ciara M. Shaver, Hong Wei Chu, R. Stokes Peebles, Dawn C. Newcomb

**Affiliations:** 1 Department of Medicine and; 2 Department of Pathology, Microbiology, and Immunology, Vanderbilt University Medical Center, Nashville, Tennessee, USA.; 3 Prevention of Organ Failure (PROOF) Centre of Excellence, University of British Columbia, Vancouver, British Columbia, Canada.; 4 National Jewish Medical Center, Denver, Colorado, USA.

**Keywords:** Inflammation, Pulmonology, Asthma, Sex hormones, T cells

## Abstract

Women have higher prevalence of asthma compared with men. In asthma, allergic airway inflammation is initiated by IL-33 signaling through ST2, leading to increased IL-4, IL-5, and IL-13 production and eosinophil infiltration. Foxp3^+^ Tregs suppress and ST2^+^ Tregs promote allergic airway inflammation. Clinical studies showed that the androgen dehydroepiandrosterone (DHEA) reduced asthma symptoms in patients, and mouse studies showed that androgen receptor (AR) signaling decreased allergic airway inflammation. Yet the impact of AR signaling on lung Tregs remains unclear. Using AR-deficient and Foxp3 fate-mapping mice, we determined that AR signaling increased Treg suppression during *Alternaria* extract (Alt Ext; allergen) challenge by stabilizing Foxp3^+^ Tregs and limiting the number of ST2^+^ ex-Tregs and IL-13^+^ Th2 cells and ex-Tregs. AR signaling also decreased Alt Ext–induced ST2^+^ Tregs in mice by limiting expression of *Gata2*, a transcription factor for ST2, and by decreasing Alt Ext–induced IL-33 production from murine airway epithelial cells. We confirmed our findings in human cells where 5α-dihydrotestosterone (DHT), an androgen, decreased IL-33–induced ST2 expression in lung Tregs and decreased Alt Ext–induced IL-33 secretion in human bronchial epithelial cells. Our findings showed that AR signaling stabilized Treg suppressive function, providing a mechanism for the sex difference in asthma.

## Graphical abstract

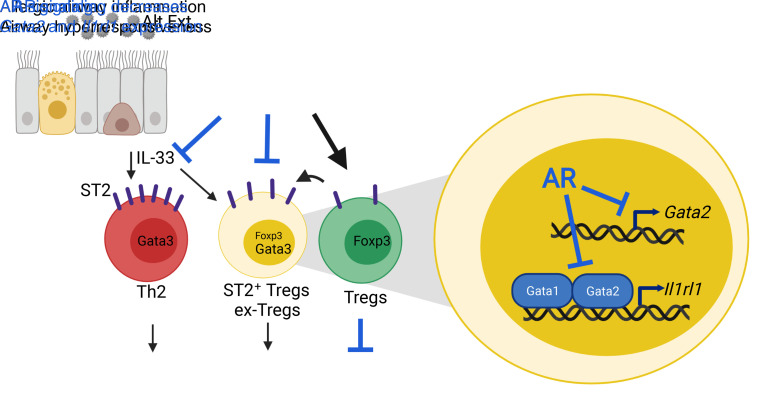

## Introduction

A sex bias exists in many autoimmune diseases and chronic inflammatory disorders, including asthma. Before puberty, males have increased asthma prevalence compared with females. After puberty, females have increased asthma prevalence compared with males. This shift in asthma prevalence coincides with increases in sex hormone levels during puberty, suggesting that sex hormones are important in asthma pathogenesis. Androgens signal through the nuclear androgen receptor (AR), and AR signaling is linked to decreased asthma symptoms and increased lung function in males and females with asthma (1–4). Increased production of dehydroepiandrosterone (DHEA), an androgen, occurs during puberty in males more than females, and higher sulfonated DHEA (DHEA-S) levels positively correlated with increases in lung function in the Severe Asthma Research Program I, II, and III cohorts (4, 5). Phase II clinical trials also showed that nebulized or slow-release oral administration of DHEA to males and females with severe asthma decreased asthma symptoms and increased lung function (4, 6). These clinical data suggest that AR signaling attenuates allergic airway inflammation.

Allergic asthma results in increased type 2 airway inflammation, eosinophil infiltration, mucus production, and airway hyperreactivity (AHR). IL-33 signaling through its receptor, ST2, is an initiator of the type 2 inflammatory cascade that leads to increased IL-4, IL-5, and IL-13 production from CD4^+^ Th2 cells, group 2 innate lymphoid cells (ILC2s), mast cells, eosinophils, and others (7–17). IL-5 is important in recruiting eosinophils into the airway. IL-4 is a critical cytokine for Th2 differentiation and supports IgE isotype switching in B cells. IL-13 increases mucus production and AHR. CD4^+^ regulatory T cells (Tregs) suppress type 2 inflammation to restore homeostasis, and Tregs are also important in establishing and maintaining tolerance to aeroallergens. Tregs decrease inflammation by producing the inhibitory cytokines IL-10 and TGF-β, and limiting proliferation of effector CD4^+^ or CD8^+^ cells, ILC2s, and granulocytes (18–23). Therefore, the presence of Tregs is imperative to maintain tolerance or restore homeostasis in response to allergen exposure, but it remains unknown how AR signaling regulates Treg responses in allergic airway inflammation.

Tregs are characterized by the expression of the transcription factor Foxp3 and can be derived in the thymus or induced from naive T cells upon activation (iTregs). Treg stability and suppressive function are maintained by Foxp3 expression (23), and mutations in Foxp3 are linked to inflammatory diseases, including Crohn’s disease, obesity, and asthma. Treg stability and suppressive function are also regulated by IL-33 signaling through ST2 depending on inflammatory state and tissue. ST2 expression on Tregs increased Treg suppressive function and reduced IFN-γ production in response to influenza infection, adipose-associated inflammation, or CD8^+^ T cell infiltration into lung adenocarcinomas (24–29). Alternatively, ST2^+^ Tregs promoted airway inflammation by increasing Gata3 expression and increasing production of IL-5 and IL-13 from Tregs during allergic airway inflammation (30, 31).

Genome-wide association studies in patients with asthma showed that *IL33* and *IL1RL1* (ST2) were the genes most associated with asthma (32–34). Bronchoalveolar lavage (BAL) levels of IL-33 inversely correlated with lung function in patients with asthma (35), and *IL33* mRNA expression was associated with more severe disease (36, 37). However, results in these studies were not analyzed based on sex, providing rationale for this study.

ST2 receptor expression is increased in asthma on CD4^+^ Th2 cells, ILC2s, mast cells, basophils, and other immune cells. ST2 expression is regulated by the Gata1, Gata2, and PU.1 transcription factors (38), and previous studies in mast cells showed that Gata2 was essential for ST2 expression (39). Further, prior studies by our laboratory and others showed a sex difference in allergic airway inflammation (40–43). Androgens signaling through AR decreased IL-33 expression in BAL fluid, type 2 airway inflammation, leukotriene production, mucus production, AHR, and mast cell degranulation (40–43). While AR signaling directly suppressed ILC2 proliferation and cytokine expression (41, 42, 44, 45), AR signaling did not directly decrease CD4^+^ Th2 cell cytokine production (40). These results suggest that AR signaling attenuates type 2 inflammation through additional, undescribed pathways. In this study, we hypothesized that AR signaling increases Treg suppressive function, leading to decreased allergic airway inflammation. Our results showed that AR signaling increased Treg suppressive function and Treg stability by limiting allergen-induced IL-33 production from epithelial cells and ST2 expression on Tregs.

## Results

### AR signaling increased Treg/Th2 ratio in the lung and decreased allergic airway inflammation.

**Figure 1:**
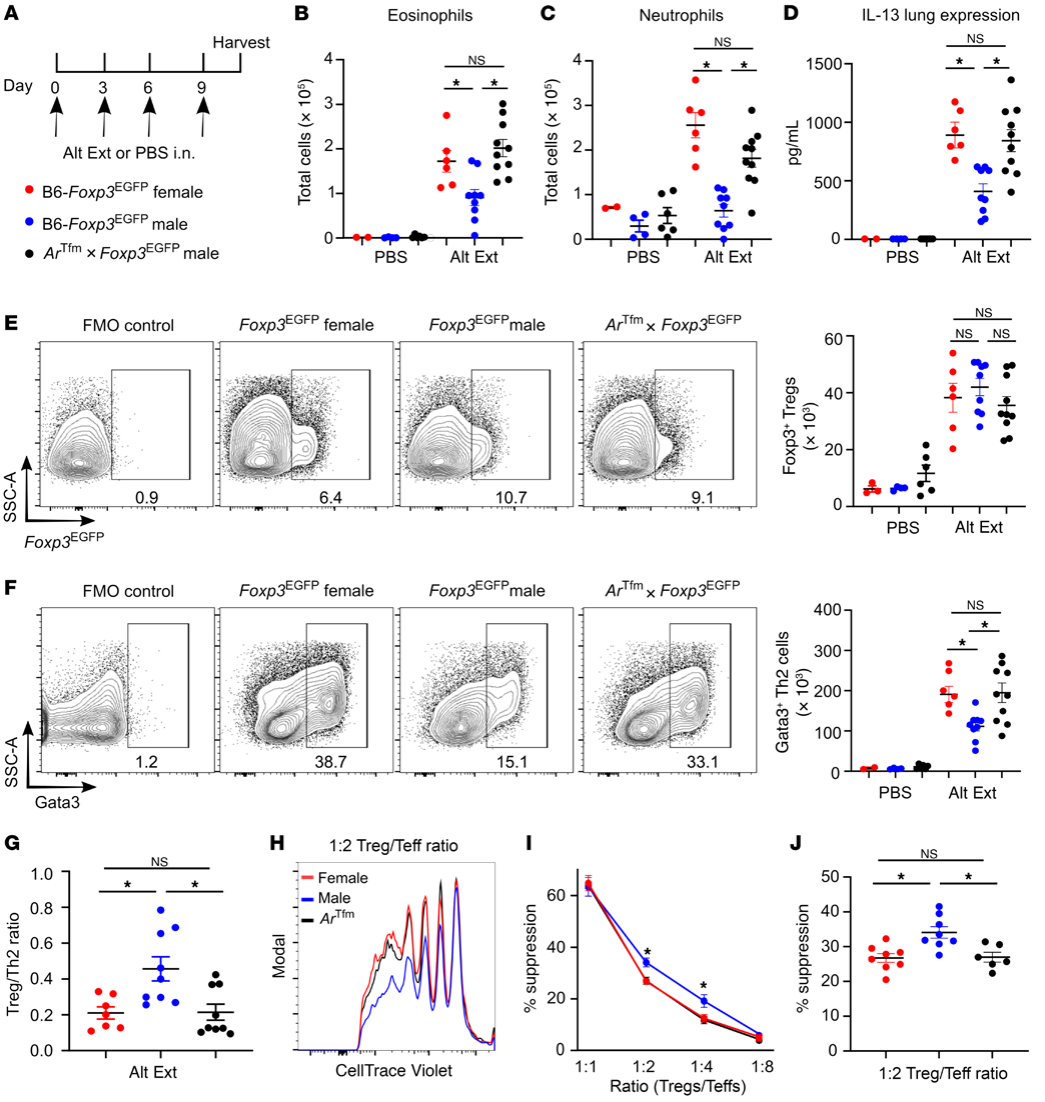
AR signaling decreases allergic airway inflammation and increases Treg suppressive function. (**A**) Experimental design of Alt Ext intranasal challenge model in B6-*Foxp3^EGFP^* female, B6-*Foxp3^EGFP^* male, and *Ar^Tfm^* *Foxp3^EGFP^* male mice. (**B** and **C**) Eosinophil and neutrophil counts in BAL fluid. (**D**) IL-13 protein expression in whole-lung homogenates. (**E**) Representative dot plots from Alt Ext–challenged mice displaying lung Tregs (Foxp3^+^CD4^+^ T cells) with quantitation to the right. (**F**) Representative dot plots from Alt Ext–challenged mice displaying lung Th2 cells (Gata3^+^CD4^+^ T cells) with quantitation to the right. In both **E** and **F**, cells were pre-gated on viable CD3^+^CD4^+^ T cells. FMO, fluorescence minus one. (**G**) Ratio of Tregs to Th2 cells in lungs of Alt Ext–challenged mice. (**B**–**G**) Data are expressed as mean ± SEM; *n =* 2–10 from 2 experiments. *P < 0.05, ANOVA with Tukey’s post hoc analysis. (**H**–**J**) Treg suppression assay using Tregs from B6-*Foxp3^EGFP^* female, B6-*Foxp3^EGFP^* male, or *Ar^Tfm^* *Foxp3^EGFP^* male mice and T effector cells from B6-*Foxp3^EGFP^* female mice. (**H**) Representative samples of T effector cell (Teff) proliferation at 1:2 Treg/T effector ratio. (**I** and **J**) Representative experiment and percentage suppression of Tregs at various ratios. (**H**–**J**) Data are expressed as mean ± SEM; *n =* 6–8 from 3 experiments. *P < 0.05, ANOVA with Tukey’s post hoc analysis.

Our previous studies showed that AR signaling attenuated *Alternaria alternata* extract–induced (Alt Ext–induced) ILC2 cytokine production as well as house dust mite–induced (HDM-induced) Th2 and IL-17A–mediated adaptive immune responses (40, 41). In this study, we focused on a new pathway of suppression in allergic airway inflammation and determined whether AR signaling increased Treg suppressive function to downregulate Alt Ext–induced allergic airway inflammation. We crossed C57BL/6 (B6) *Foxp3^EGFP^* male mice with *Ar^Tfm^* heterozygous female mice to generate *Ar^Tfm^* *Foxp3^EGFP^* male mice. *Ar^Tfm^* male mice have a mutation in the AR, preventing the AR from responding to androgens. To induce allergic airway inflammation, B6-*Foxp3^EGFP^* female, B6-*Foxp3^EGFP^* male, and *Ar^Tfm^* *Foxp3^EGFP^* male mice were intranasally challenged with Alt Ext or vehicle control (PBS) once every 3 days for 4 total challenges (Figure 1A). Similar to our previous findings with HDM (40), Alt Ext–challenged B6-*Foxp3^EGFP^* male mice had decreased eosinophils and neutrophils in BAL fluid as well as decreased IL-13 production in whole-lung homogenates compared with B6-*Foxp3^EGFP^* female and *Ar^Tfm^* *Foxp3^EGFP^* male mice (Figure 1, B–D, and ref. 40). Next, we quantitated the numbers of Tregs (Foxp3eGFP^+^CD4^+^CD3^+^ cells) and Th2 cells (Gata3^+^CD4^+^CD3^+^ cells) in the lung. Alt Ext–challenged B6-*Foxp3^EGFP^* male mice had similar numbers of Tregs but decreased numbers of Th2 cells compared with Alt Ext–challenged B6-*Foxp3^EGFP^* female and *Ar^Tfm^* *Foxp3^EGFP^* male mice (Figure 1, E and F, and Supplemental Figure 1; supplemental material available online with this article; https://doi.org/10.1172/JCI153397DS1). Therefore, we determined the ratio of Tregs to Th2 cells in the lung. Alt Ext–challenged B6-*Foxp3^EGFP^* male mice had increased Treg/Th2 ratio compared with B6-*Foxp3^EGFP^* female and *Ar^Tfm^* *Foxp3^EGFP^* male mice (Figure 1G), showing that AR signaling increased the proportion of Tregs relative to Th2 cells in response to allergen challenge. We confirmed these findings using a model in which androgens were drastically reduced in WT male mice through castration (gonadectomy). In this model, hormonally intact, WT male mice had decreased airway inflammation and increased Treg/Th2 ratio compared with gonadectomized male mice that lacked androgens (Supplemental Figure 2). Combined, these data suggest that AR signaling increased Treg numbers relative to Th2 cells and attenuated allergen-induced type 2 inflammation.

### AR signaling increased Treg suppressive function.

Treg suppressive function can be determined by the ability of a Treg to prevent proliferation of a CD4^+^ effector cell. We hypothesized that AR signaling increases Treg suppressive function. To test this hypothesis, we sorted splenic CD4^+^ Tregs from B6-*Foxp3^EGFP^* male, B6-*Foxp3^EGFP^* female, and *Ar^Tfm^* *Foxp3^EGFP^* male mice and cocultured these Tregs at various ratios with CellTrace Violet–labeled CD4^+^ T effectors and irradiated splenocytes from B6-*Foxp3^EGFP^* female mice (46). Tregs from B6-*Foxp3^EGFP^* male mice suppressed proliferation of CD4^+^ T effector cells to a greater degree than Tregs from B6-*Foxp3^EGFP^* female and *Ar^Tfm^* *Foxp3^EGFP^* male mice (Figure 1, H–J). These data suggest that AR signaling promotes Treg suppressive function.

**Figure 2:**
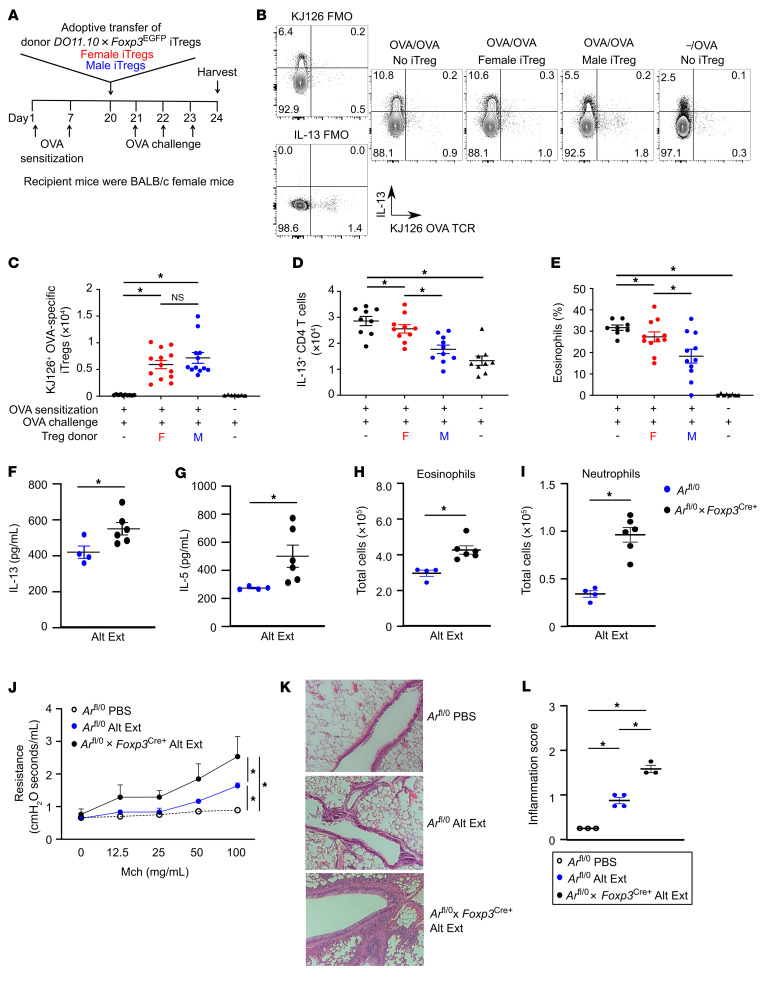
AR signaling increases the suppressive function of Tregs during ongoing allergic airway inflammation. (**A**) Experimental model for OVA-induced allergic airway inflammation with adoptive transfer of OVA-specific iTregs from *DO11.10 Foxp3^EGFP^* male or female mice and T effectors from *DO11.10* female mice. (**B**) Representative dot plots showing OVA-specific iTregs and IL-13 production in lungs of recipient mice. Cells were pre-gated on viable, CD3^+^CD4^+^ cells. (**C** and **D**) Quantification of OVA-specific iTregs and IL-13^+^CD4^+^ T cells in lungs of recipient mice. (**E**) Percentage of eosinophils in BAL fluid from recipient mice. (**B**–**E**) Data are expressed as mean ± SEM; *n =* 9–11 from 2 separate experiments. *P < 0.05, ANOVA with Tukey’s post hoc analysis. (**F**–**I**) *Ar^fl/0^* and *Ar^fl/0^* *Foxp3^Cre+^* male mice were intranasally challenged with Alt Ext, and BAL and lungs were harvested 1 day after the last challenge. (**F** and **G**) Whole-lung IL-13 and IL-5 levels. (**H** and **I**) BAL eosinophil and neutrophil cell counts. Data are expressed as mean ± SEM; *n =* 4–6. *P < 0.05, Student’s *t* test. (**J**–**L**) *Ar^fl/0^* and *Ar^fl/0^* *Foxp3^Cre+^* male mice were intranasally challenged with Alt Ext or PBS, and lungs were harvested 2 days after the last Alt Ext or PBS challenge for airway physiology or histology. (**J**) Airway resistance to increasing concentrations of nebulized methacholine (Mch) was determined. (**K** and **L**) Airway inflammation. Whole lungs were stained with H&E, and airway inflammation was scored. Original magnification of images, ×10. (**J**–**L**) Data are expressed as mean ± SEM; *n =* 3–4. *P < 0.05, ANOVA with repeated analysis and Tukey’s post hoc test for **J** and ANOVA with Tukey’s post hoc analysis for **L**.

We next evaluated whether induced Tregs (iTregs) from male mice had increased suppressive function in vivo compared with iTregs from female mice. To conduct this experiment, we crossed *DO11.10* BALB/c mice that have an OVA-specific CD4^+^ T cell receptor with BALB/c *Foxp3^EGFP^* mice to generate *DO11.10* *Foxp3^EGFP^* mice. As observed in B6 mice, Tregs from male *DO11.10* BALB/c mice were more suppressive than Tregs from female *DO11.10* BALB/c mice in vitro (Supplemental Figure 3), providing feasibility for using these Tregs for adoptive transfer experiments to evaluate suppressive function in vivo. On days 0 and 7, BALB/c female mice were sensitized with an intraperitoneal injection of OVA/aluminum hydroxide (Figure 2A). On day 20, iTregs from female and male *DO11.10* *Foxp3^EGFP^* mice were adoptively transferred into recipient BALB/c female mice. Recipient BALB/c female mice were then challenged with nebulized OVA (days 21–23), and lungs and BAL fluid were harvested 24 hours later (day 24). Similar numbers of iTregs from *DO11.10 Foxp3^EGFP^* male and female mice migrated to lung (Figure 2, B and C). However, recipient mice that had received iTregs from *DO11.10* *Foxp3^EGFP^* male mice had decreased IL-13^+^ Th2 cells and BAL eosinophils compared with recipient mice that had received no iTregs or recipient mice that had received iTregs from *DO11.10* *Foxp3^EGFP^* female mice (Figure 2, B, D, and E). These data show that male Tregs suppressed OVA-induced allergic airway inflammation to a greater degree than female Tregs in vivo.

Next, we determined whether AR signaling in Tregs was imperative for suppressive allergic airway inflammation. We crossed *Ar^floxed^* mice and *Foxp3^eYFP-Cre^* mice to generate mice with AR-deficient Tregs (*Ar^fl/0^* *Foxp3^Cre+^* for males) or littermate male controls (*Ar^fl/0^* mice). PCR determined that AR expression was lost in Tregs of *Foxp3^Cre+^* mice (Supplemental Figure 4); therefore we challenged *Ar^fl/0^* *Foxp3^Cre+^* or *Ar^fl/0^* male mice with Alt Ext as shown in Figure 1A. BAL fluid and lungs were harvested 24 hours after Alt Ext challenge, and we determined that mice with AR-deficient Tregs (*Ar^fl/0^* *Foxp3^Cre+^* mice) had significantly increased Alt Ext–induced IL-13 and IL-5 production in the lungs and infiltration of eosinophils and neutrophils in the BAL fluid compared with *Ar^fl/0^* male mice with normal AR signaling in Tregs (Figure 2, F–I). In separate experiments, Alt Ext–challenged *Ar^fl/0^* *Foxp3^Cre+^* or *Ar^fl/0^* male mice were harvested on day 11 to determine AHR to methacholine and to examine inflammation by histopathology. Loss of AR signaling in Tregs in the *Ar^fl/0^* *Foxp3^Cre+^* male mice increased AHR as measured by increased airway resistance in response to nebulized methacholine (Figure 2J). Further, *Ar^fl/0^* *Foxp3^Cre+^* male mice had increased Alt Ext–induced airway inflammation as determined by H&E staining compared with *Ar^fl/0^* male mice (Figure 2, K and L), but no increases in mucus production determined by periodic acid–Schiff staining (data not shown). These data show that AR signaling in Tregs increases Treg suppressive function to decrease allergic airway inflammation and AHR.

### AR signaling increased Treg stability.

**Figure 3:**
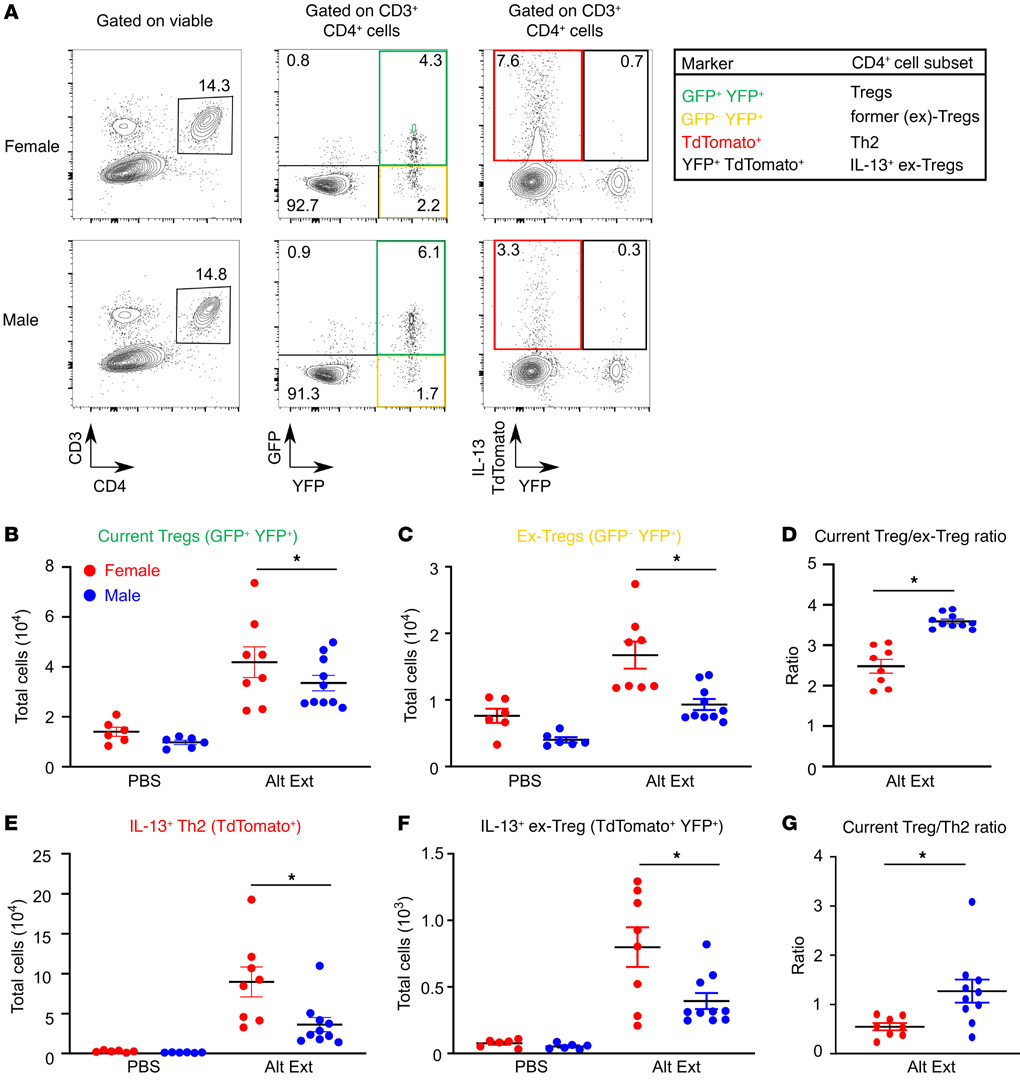
Tregs from Alt Ext–challenged male mice are more stable than Tregs from female mice. *Foxp3^GFP/YFP^Il13^TdTomato^* female and male mice underwent Alt Ext protocol. (**A**) Representative dot plots of lung CD4^+^ T cells in Alt Ext–challenged mice showing current Tregs, ex-Tregs, Th2 cells, and IL-13^+^ ex-Tregs. (**B** and **C**) Numbers of current Tregs and ex-Tregs in lungs of mice. (**D**) Ratio of current Tregs to ex-Tregs. (**E** and **F**) Numbers of IL-13^+^ Th2 cells and IL-13^+^ ex-Tregs in lungs of mice. (**G**) Ratio of current Tregs to Th2 cells. Data are mean ± SEM; *n =* 6–10 from 2 separate experiments. *P < 0.05, ANOVA with Tukey’s post hoc analysis (**B**–**F**), Student’s *t* test (**G**).

Results from the previous 2 experiments indicated a sex difference in Treg suppressive function and that AR plays a role in Treg suppressive function. The identity and suppressive function of Tregs are dependent on maintaining Foxp3 expression (23). Therefore, we next determined whether Tregs from male mice had increased Foxp3 stability compared with Tregs from female mice during allergic airway inflammation. To conduct this experiment, we used Foxp3 fate-mapping BALB/c mice that were crossed to *Il13^TdTomato^* mice to generate *Foxp3*^EGFP-Cre^ *Rosa26^YFP/YFP^* *Il13^TdTomato^* mice that will be referred to as *Foxp3^GFP/YFP^* *Il13^TdTomato^* mice. These mice provide the ability to identify current Tregs (GFP^+^YFP^+^), CD4^+^ Th2 cells (GFP^–^YFP^–^TdTomato^+^), and CD4^+^ T cells that previously expressed Foxp3 but currently do not express Foxp3 (ex-Tregs, GFP^–^YFP^+^TdTomato^+/–^). Further, these mice provide the opportunity to determine Treg stability and the conversion into IL-13–producing T effector cells. Using the Alt Ext protocol, we determined that male mice had decreased infiltration of eosinophils and neutrophils in BAL fluid (Supplemental Figure 5, A and B) and decreased IL-13^+^ Th2 cells in the lungs (Figure 3, A and E), similar to our results in B6 mice. Consistent with our previous experiments, there were no differences in Treg numbers between males and females. However, male mice had a significant decrease in ex-Tregs and IL-13^+^ ex-Tregs after Alt Ext challenge and similar numbers of current Tregs compared with female mice (Figure 3, A–C, E, and F). Further, both the current Treg/ex-Treg ratio and the Treg/Th2 ratio were higher in males (Figure 3, D and G). These data suggested that Tregs from males were more stable, providing a mechanism for the sex difference in Treg suppressive function during allergic airway inflammation and the Treg/Th2 ratio.

**Figure 4:**
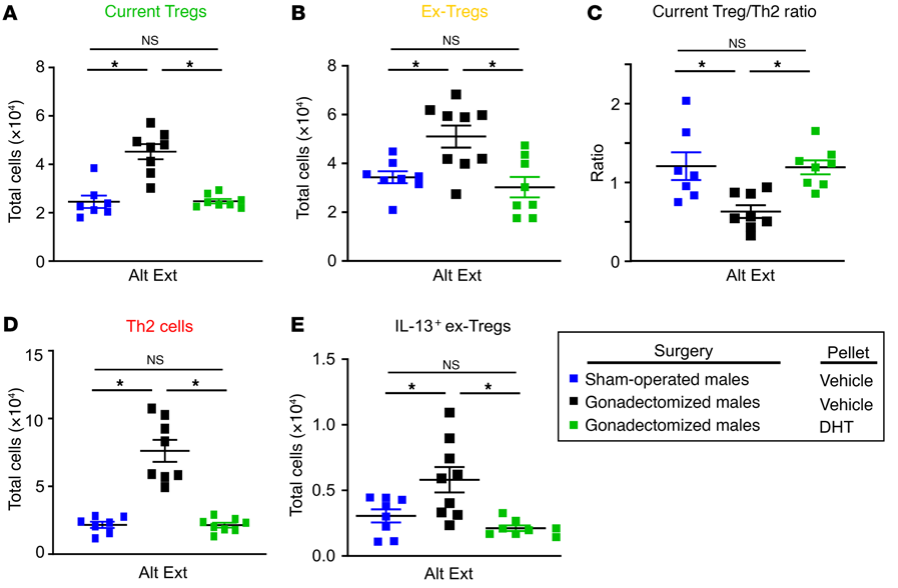
AR signaling improves Treg stability during ongoing allergic airway inflammation. *Foxp3^GFP/YFP^Il13^TdTomato^* male mice underwent gonadectomy or sham operation at 3–4 weeks of age. At 8 weeks old, 5α-DHT or vehicle slow-release pellets were subcutaneously placed into mice. At 11 weeks old, mice underwent Alt Ext protocol. (**A** and **B**) Numbers of current Tregs and ex-Tregs in lungs of mice. (**C**) Ratio of current Tregs to Th2 cells. (**D**) Numbers of IL-13^+^ Th2 cells in lungs of mice. (**E**) Numbers of IL-13^+^ ex-Tregs in lungs of mice. Data are mean ± SEM; *n =* 7–9 from 2 separate experiments. *P < 0.05, ANOVA with Tukey’s post hoc analysis.

Figure 3 shows a sex difference in Treg stability, but these data did not determine whether AR signaling increased Treg stability. Therefore, we conducted an additional experiment in which *Foxp3^GFP/YFP^ Il13^TdTomato^* male mice underwent a gonadectomy or sham surgery at age 3–4 weeks, prior to puberty. At 8 weeks of age, a slow-release pellet containing 5α-dihydrotestosterone (DHT) or vehicle was subcutaneously implanted into gonadectomized or sham-operated *Foxp3^GFP/YFP^* *Il13^TdTomato^* male mice. At 11 weeks, these mice were intranasally challenged with Alt Ext, and lungs were harvested on day 10 to determine the number of current Tregs, Th2 cells, ex-Tregs, and IL-13^+^ ex-Tregs by flow cytometry, eosinophil and neutrophil infiltration into BAL fluid, and whole-lung IL-5 and IL-13 protein expression. 5α-DHT administration decreased eosinophils and neutrophils in BAL fluid as well as whole-lung IL-5 and IL-13 production (Supplemental Figure 5, C–F). Hormonally intact male mice and gonadectomized male mice given 5α-DHT also had significantly decreased numbers of Th2 cells, ex-Tregs, and IL-13^+^ ex-Tregs compared with gonadectomized male mice given vehicle (Figure 4, A–E). Further, 5α-DHT administration to gonadectomized male mice increased the Treg/Th2 ratio in the lungs of Alt Ext–challenged mice. These data show that 5α-DHT increased Treg stability during allergic airway inflammation.

### AR signaling decreased IL-33 expression in airway epithelial cells and attenuated allergen-induced ST2^+^ Tregs.

**Figure 5:**
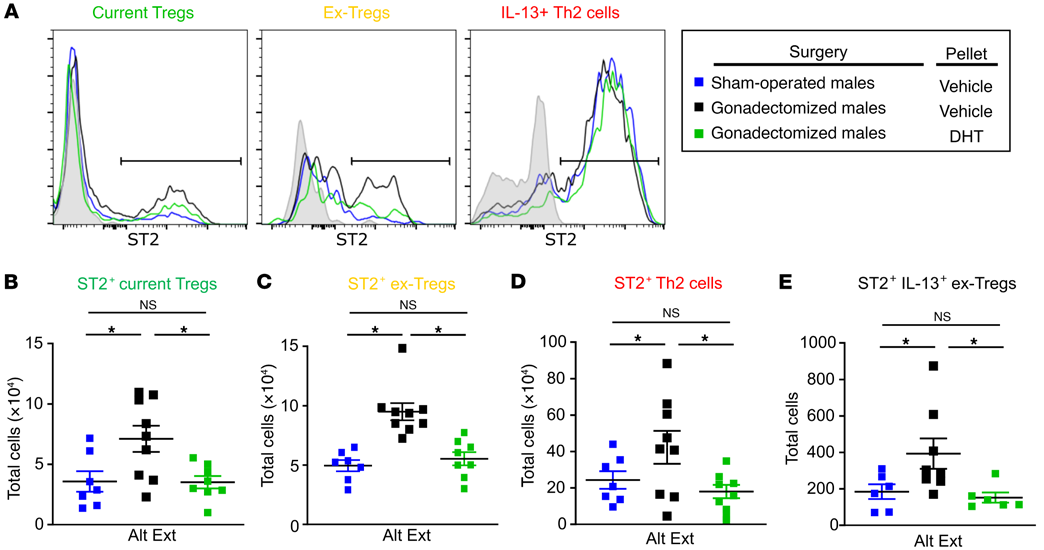
AR signaling decreases ST2^+^ Tregs after allergen challenge. *Foxp3^GFP/YFP^Il13^TdTomato^* male mice underwent gonadectomy or sham operation at 3–4 weeks of age. At 8 weeks old, 5α-DHT or vehicle slow-release pellets were subcutaneously placed into mice. After 3 weeks, mice underwent Alt Ext protocol. (**A**–**E**) Histograms and quantification of ST2-expressing Tregs, ex-Tregs, Th2 cells, and IL-13^+^ ex-Tregs. Data are mean ± SEM; *n =* 7–9 from 2 separate experiments. *P < 0.05, ANOVA with Tukey’s post hoc analysis.

We showed that AR signaling increased Treg suppressive function during allergic airway inflammation, but the mechanism of how AR signaling maintained Foxp3 expression in Tregs remained unclear. Prior studies showed that ST2^+^ Tregs increased expression of Gata3, a transcription factor that is essential for IL-4, IL-5, and IL-13 production, and limited Foxp3 expression and Treg suppressive function (30, 31). Further, our previous results showed that AR signaling decreased ST2 expression on lung ILC2s (41). Therefore, AR signaling may decrease ST2 expression on Tregs, providing a mechanism for decreasing allergic airway inflammation. To determine whether AR signaling reduced ST2 expression on Tregs, we next quantified the number of ST2^+^ Tregs, ex-Tregs, and Th2 cells in Alt Ext–challenged male and female mice from Figure 3. Male mice had decreased ST2-expressing Tregs, ex-Tregs, and Th2 cells compared with female mice, but no differences in ST2 mean fluorescence intensity (MFI) were determined in Tregs and ex-Tregs of male and female mice (Supplemental Figure 6). Next, we quantified ST2 expression in Tregs, ex-Tregs, Th2 cells, and IL-13^+^ ex-Tregs from the lungs of Alt Ext–challenged, hormonally intact male and gonadectomized male mice. AR signaling decreased ST2 expression on all cell types, as hormonally intact male mice and gonadectomized male mice given 5α-DHT had decreased ST2^+^ Tregs, ST2^+^ ex-Tregs, ST2^+^ Th2 cells, and ST2^+^IL-13^+^ ex-Tregs compared with gonadectomized male mice (Figure 5, A–E). These data show that AR signaling downregulated the number of ST2^+^ Tregs during allergic airway inflammation.

**Figure 6:**
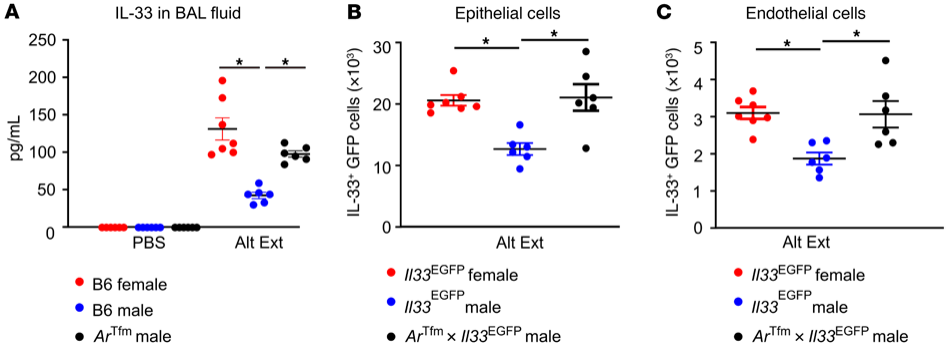
AR signaling decreases IL-33 production and secretion in mice. (**A**) B6 female, B6 male, and *Ar^Tfm^* male mice were challenged with Alt Ext or PBS, and BAL fluid was collected 1 hour after the final Alt Ext challenge. IL-33 production was examined by ELISA. (**B** and **C**) *Il33^EGFP^* female, *Il33^EGFP^* male, and *Ar^Tfm^* *Il33^EGFP^* male mice underwent the Alt Ext protocol, and IL-33 (GFP)^+^ epithelial cells and endothelial cells were determined in the lung by flow cytometry. Data are mean ± SEM; *n =* 6 from 2 separate experiments. *P < 0.05, ANOVA with Tukey’s post hoc analysis.

ST2 expression on T cells and ILC2s is increased after IL-33 stimulation, and IL-33 is released quickly in response to protease-containing allergens (41). Therefore, we administered Alt Ext or PBS to B6 male, B6 female, and *Ar^Tfm^* male mice, and BAL fluid was collected 1 hour after the last challenge. *Ar^Tfm^* male mice, lacking AR signaling, had increased IL-33 protein expression compared with B6 male mice (Figure 6A). To determine the cellular sources of IL-33, we next challenged *Il33^EGFP^* male, *Il33^EGFP^* female, and *Ar^Tfm^* *Il33^EGFP^* male mice with Alt Ext and conducted flow cytometry. AR signaling attenuated the numbers of IL-33^+^ lung epithelial cells (CD45^–^EpCAM^+^) and lung endothelial cells (CD45^–^CD146^+^) (Figure 6, B and C, and Supplemental Figure 7). IL-33^+^ mast cells and macrophages were also quantitated, and no significant differences between *Il33^EGFP^* male and *Ar^Tfm^* *Il33^EGFP^* male mice were determined (data not shown). These data show that AR signaling decreased IL-33 production, providing an indirect mechanism for decreased numbers of ST2^+^ Tregs.

**Figure 7:**
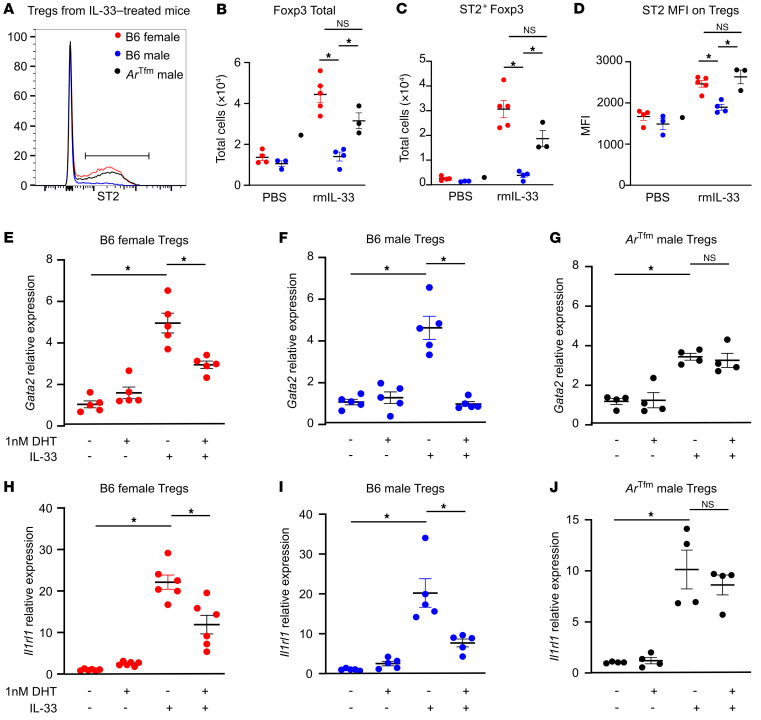
AR signaling decreases IL-33–induced ST2 expression on Tregs by decreasing *Gata2* expression. B6 female, B6 male, and *Ar^Tfm^* male mice were given rmIL-33 (300 ng) or vehicle (“–”, PBS) every 3 days for 9 days total. On day 10, lungs were harvested for analysis. (**A**) ST2 expression on lung Tregs. (**B** and **C**) Quantification of Tregs and ST2^+^ Tregs in PBS and rmIL-33 groups of mice. (**D**) ST2 MFI on Tregs. (**E**–**J**) Splenic Tregs were isolated from mice and restimulated in the presence of 5α-DHT (1 nM) and/or rmIL-33 (100 ng/mL) for 3 days. *Gata2* and *Il1rl1* (ST2) relative expression to vehicle was determined based on expression of *Gapdh*. Data are mean ± SEM; *n =* 3–5. *P < 0.05, ANOVA with Tukey’s post hoc analysis.

Next, we hypothesized that AR signaling decreases ST2 expression on Tregs and Th2 cells. To test this hypothesis, we administered recombinant mouse IL-33 (rmIL-33) to B6-*Foxp3^EGFP^* female, B6-*Foxp3^EGFP^* male, and *Ar^Tfm^* *Foxp3^EGFP^* male mice using the Alt Ext protocol to ensure that each group of mice had similar IL-33 levels in the lung. rmIL-33 increased the number of ST2^+^ Tregs as well as ST2 MFI compared with PBS control, and AR signaling decreased the number of ST2^+^ Tregs and ST2 MFI (Figure 7, A–D). Previous studies reported that ST2 expression on Tregs decreased Bcl6 expression, allowing for increased Gata3 expression and production of IL-4, IL-5, and IL-13 (31). Based on these findings, we also examined the percentage of Bcl6^+^ Tregs after rmIL-33 administration and determined that Tregs from B6 *Foxp3^EGFP^* male mice had increased Bcl6 expression compared with female B6 *Foxp3^EGFP^* female and *Ar^Tfm^* *Foxp3^EGFP^* male mice (Supplemental Figure 8).

These data suggested that AR signaling had a direct effect on ST2 expression in Tregs. ST2 expression is driven by increased activation of the transcription factors Gata1, Gata2, and PU.1 (47). Therefore, we next determined the relative expression of these transcription factors as well as *Il1rl1* (ST2) by quantitative PCR in mouse Tregs that were stimulated in vitro with IL-2, IL-33, and/or 5α-DHT. As expected, *Gata2* and *Il1rl1* were significantly increased by IL-33 in Tregs from male mice (Figure 7, E–J). Preincubation with 5α-DHT significantly decreased *Gata2* and *Il1rl1* expression in B6 female and B6 male Tregs, but not in *Ar^Tfm^* male Tregs (Figure 7, E–J). Preincubation with 5α-DHT had no effect on *Gata1* expression, and *Pu1* expression was not detected in Tregs (data not shown). These data confirmed that AR signaling decreased expression of *Gata2*, an essential transcription factor for ST2 expression in Tregs.

### 5α-DHT decreased human airway epithelial IL-33 release and decreased ST2 expression on human Tregs.

**Figure 8:**
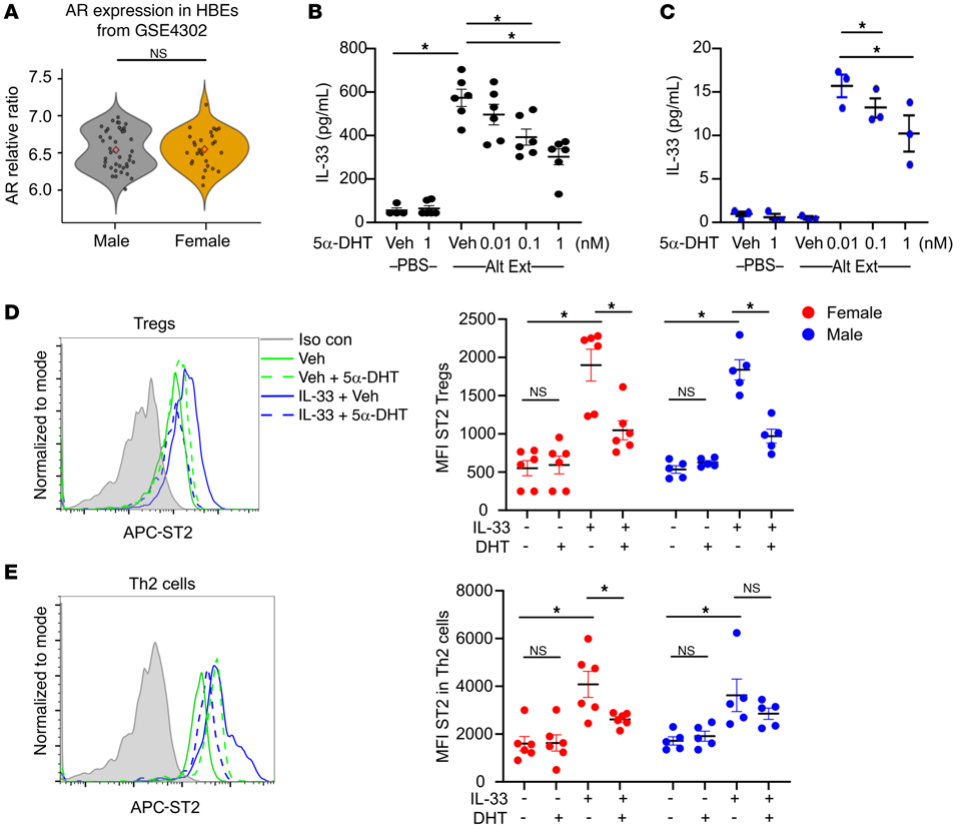
5α-DHT decreases IL-33 secretion on human airway epithelial cells and reduces ST2 expression on human lung Tregs. (**A**) *Ar* expression in human bronchial epithelial cells (HBEs) from male and female subjects (control and asthma participants are combined) in the GEO GSE4302 study with statistical analysis by Student’s *t* test. (**B** and **C**) hBE33 cells or primary, differentiated HBEs were treated for 24 hours with vehicle (Veh) or 5α-DHT (0–1 nM) and stimulated with Alt Ext (30 μg/mL) for 1 hour. IL-33 was measured in supernatants by ELISA. hBE33 cells, *n =* 4 from 2 separate experiments; HBEs from male asthmatic individuals, *n =* 3 donors. Data are mean ± SEM. *P < 0.05, ANOVA with Tukey’s post hoc analysis. (**D** and **E**) CD45^+^ cells isolated from excised human lungs were stimulated with anti-CD3 and anti-CD28 in the presence of 5α-DHT (1 nM), IL-33 (40 ng/mL), and/or vehicle for 24 hours. Tregs (CD3^+^CD4^+^Foxp3^+^) and Th2 cells (CD3^+^CD4^+^Gata3^+^) were pre-gated, and ST2 MFI was determined for each group. Histogram of ST2 in Tregs or Th2 cells is shown. Data are mean ± SEM; *n =* 6 female and 5 male donors. *P < 0.05, ANOVA with Tukey’s post hoc analysis.

Based on our mouse data in Figure 6 showing that AR signaling decreased IL-33 release, we determined whether testosterone or androgens decreased Alt Ext–induced IL-33 secretion from human bronchial epithelial cells (HBEs). Using the Gene Expression Omnibus (GEO) GSE4302 data set and confirming in GSE63142, GSE43696, and GSE41861 data sets, we determined that *AR* expression levels in HBEs from men and women are similar (Figure 8A and refs. 48–52). Further, no sex differences were seen in asthmatic-only samples (data not shown). Since *AR* was expressed at similar levels in male and female HBEs, we next wanted to determine whether 5α-DHT decreased allergen-induced IL-33 secretion. We used an HBE cell line that was engineered to constitutively express IL-33, hBE33 cells. hBE33 cells were preincubated with 5α-DHT (0.01–1 nM) or methanol (vehicle) for 24 hours, and then cells were challenged with Alt Ext (30 μg/mL) for 1 hour. Culture supernatants were collected, and 5α-DHT decreased IL-33 release in response to Alt Ext (Figure 8B). Cell lines may not exhibit the same properties as primary HBEs, and so we repeated these experiments in primary HBEs from 3 male asthmatic individuals. Pretreatment with 5α-DHT (1 nM) decreased IL-33 secretion from these primary HBEs (Figure 8C). These data show that AR signaling decreases Alt Ext–induced IL-33 release in mice as well as in human epithelial cells and provide a mechanism for AR signaling decreasing ST2 expression on Tregs as well as CD4^+^ Th2 cells and ILC2s.

Next, we wanted to determine whether 5α-DHT decreased ST2 expression on IL-33–stimulated human lung Tregs and Th2 cells. CD45^+^ cells were isolated from excised human lungs not suitable for transplant and restimulated in the presence of IL-33, 5α-DHT (1 nM), and/or vehicle. ST2 expression on Tregs and Th2 cells was then determined by flow cytometry staining. In cells from females and males, IL-33 increased ST2 expression on Tregs, and this was attenuated in the presence of 5α-DHT (Figure 8D). Further, IL-33 increased ST2 expression on Th2 cells from females and males, but 5α-DHT attenuated IL-33–induced ST2 expression only in the female Th2 cells (Figure 8E). These data support that AR signaling decreases IL-33–mediated upregulation of ST2 expression on Tregs as well as Th2 cells from human lungs.

## Discussion

This study determined a mechanism by which AR signaling increased Treg suppressive function to decrease airway inflammation associated with asthma. Clinical studies have reported that androgens and AR signaling decreased asthma symptoms and increased lung function (1–4), and patients with androgen insensitivity syndrome had a greater risk of asthma compared with control patients (53). Further, DHEA administration and the rate of DHEA conversion into downstream androgens increased glucocorticoid responsiveness and/or lung function in patients with asthma (5, 6, 23). These data provide clinical relevance for determining how AR signaling attenuates airway inflammation in asthma. Yet, how AR signaling decreased airway inflammation remained unclear.

While it was previously reported that AR signaling decreased type 2 allergic airway inflammation, AHR, and mucus production (40–43), findings from this study determined that AR signaling stabilizes Treg suppressive function as an additional mechanism to decrease allergic airway inflammation. It has long been established that Foxp3 expression is associated with the suppressive function of Tregs (54). Treg stability requires maintaining Foxp3 expression and limiting the expression of effector cytokines, including type 2 cytokines, during allergic airway inflammation (54). Using Foxp3 fate-mapping mice crossed to *Il13^TdTomato^* reporter mice, we were able to track how androgens modified Treg stability or plasticity during allergic airway inflammation. Our data showed that AR signaling maintained suppressive capacity in Tregs in vitro. Further, we showed that AR signaling in Tregs decreased allergic airway inflammation and AHR, providing additional mechanisms for how AR signaling attenuates asthma pathogenesis.

Our study uncovered a mechanism for how AR signaling was maintaining Treg suppressive function during allergic airway inflammation. IL-33 increases ST2 expression on T cells, including Tregs, during allergic airway inflammation. ST2^+^ Tregs were recently shown to increase allergic airway inflammation by upregulating expression of Gata3, a transcription factor that is essential for IL-4, IL-5, and IL-13 production (31). Typically in Tregs, Bcl6 promotes Foxp3 expression and represses Gata3 expression, prompting suppressive Tregs. However, IL-33 signaling through ST2 increased expression of Blimp1, a negative regulator of Bcl6, leading to increased Gata3 expression and production of IL-4, IL-5, and IL-13 from ST2^+^ Tregs (31). Our previous studies showed that AR signaling attenuated ST2 expression on ILC2s (55), and therefore we explored whether AR signaling reduced ST2 expression on Tregs as a mechanism to stabilize Treg suppressive function. We determined that AR signaling decreased ST2 expression on all Tregs as well as decreased the shift of Tregs to ex-Tregs and IL-13^+^ ex-Tregs. We also determined that AR signaling maintained Bcl6 expression in Tregs after rmIL-33 exposure, providing additional evidence that AR signaling limits IL-33/ST2 signaling in Tregs as a mechanism to maintain Treg suppressive function.

ST2 expression on T cells, including Tregs, and other immune cells is increased in a positive-feedback mechanism with IL-33/ST2 signaling further upregulating ST2 (56). We determined that AR signaling decreased IL-33 production in epithelial cells from humans and mice after allergen exposure, providing an indirect or extrinsic mechanism to limit ST2 expression on Tregs. Further, we showed that androgens and AR signaling limit IL-33 production, leading to a reduction in ST2 expression on Th2 cells and Tregs and decreased allergic airway inflammation.

Lung IL-33 is predominantly expressed in endothelial and epithelial cells in humans and mice (57, 58). Under normal conditions, full-length IL-33, the primary form of IL-33 (59), is bound to histones in the chromatin and sequestered in the nucleus to prevent an unprovoked inflammatory response (60). AR signaling limited production of IL-33 in airway epithelial cells and endothelial cells of mice as well as limited its release in HBEs challenged with allergen. Since IL-33 is sequestered in the nucleus by binding to histones, it is possible that AR signaling may increase IL-33 binding to histone to slow or reduce the release of IL-33 from this nucleus. IL-33 can also bind to soluble ST2, a decoy receptor, that prevents downstream activation (61). Therefore, AR signaling increasing soluble ST2 is another potential mechanism to reduce active IL-33 secretion. Using Alt Ext–challenged B6 male and *Ar^Tfm^* male mice, we determined that soluble ST2 in whole-lung homogenates was similar between B6 male and *Ar^Tfm^* male mice (0.59 ± 0.03 ng/mL in B6 males and 0.61 ± 0.02 ng/mL in *Ar^Tfm^* males), suggesting that this was not how AR regulated IL-33 release. All together, these data show that AR signaling attenuated IL-33 release from human and murine epithelial cells as an extrinsic mechanism to limit ST2^+^ Tregs and decrease allergic airway inflammation. Additional studies are needed to determine how AR signaling attenuates IL-33 production and release in airway epithelial and endothelial cells.

Our findings demonstrate that AR signaling decreased ST2 expression on Tregs through an intrinsic mechanism as well. Transcription of *Il1rl1* is regulated by the transcription factors Gata1, Gata2, and PU.1 (38). AR signaling decreased expression of *Gata2*, providing an intrinsic mechanism for limiting ST2^+^ Tregs. A TRANSFAC (GeneXplain) database search previously showed the AR consensus binding sequencing in the promoter regions of *Gata2* and *Il1rl1*, but not *Gata1* (62, 63). AR signaling was also recently shown in Sertoli cells to decrease Gata2 protein expression (64). However, in the prostate cancer cell lines, Gata2 is one of the transcription factors that regulates *Ar* expression (65, 66). Therefore, in our study we determined that 5α-DHT decreased *Gata2* and *Il1rl1* (ST2) expression in IL-33–stimulated Tregs and that there was no difference in *Ar* expression in vehicle- and 5α-DHT–treated cells. We postulate that in Tregs, AR directly binds to the promoter region of *Gata2* and *Il1rl1* to negatively regulate transcription.

Other pathways, independent of ST2, may also be responsible for AR signaling–mediated intrinsic maintenance of Treg suppressive function and stability and were not the focus of this study. Prostacyclin was recently shown to maintain Treg suppressive function by limiting the β-catenin pathway to decrease allergic airway inflammation, providing rationale for determining how AR signaling regulates β-catenin pathways (67). Further, AR signaling is known to epigenetically regulate human Tregs, as the androgen response element increased H4 acetylation, but not CpG methylation, to increase *Foxp3* expression in vitro in human Tregs (68). Several histone acetyltransferases, including p300, HDAC7, HDAC9, SIRT1, and TIP60, have been shown to increase histone acetylation in the *Foxp3* locus (69). Therefore, AR signaling may increase histone acetylation in the *Foxp3* locus of Tregs by increasing expression of or colocalizing with histone acetyltransferases. Finally, recent studies also showed that epigenetic regulation or alterations in the Notch and Wnt signaling pathways are also important in Treg function, and that Notch4 signaling promoted type 2 cytokine expression in Tregs (31, 70). So, AR signaling may interact with the Notch and Wnt signaling pathways to improve Treg stability during allergic airway inflammation.

While ST2^+^ Tregs promote type 2 allergic airway inflammation, ST2^+^ Tregs suppress IFN-γ–mediated inflammation in adipose tissue, during influenza infections, and in lung adenocarcinoma (24–29). In adipose tissue, AR signaling increased IL-33 production from stromal cells in adipose tissue, resulting in increased ST2^+^ Tregs and decreased M1 macrophages and CD8^+^ T cells (26). Type 2 immune cells, M2 macrophages, ILC2s, and ST2^+^ Tregs restore homeostasis and decrease IFN-γ–mediated inflammation in inflamed, obese adipose tissue and in response to influenza. In tumor development, CD8^+^ infiltrating T cells are imperative for destroying tumor cells, and ST2^+^ Tregs suppress the infiltration of these CD8^+^ T cells (27). Therefore, ST2^+^ Tregs have diverse functions based on the type of inflammatory response present, and it will be interesting for future studies to explore how AR signaling and estrogen signaling modify ST2^+^ Treg responses in other diseases with a sex bias.

In summary, our results show that AR signaling increases Treg suppressive function by limiting ST2^+^ Tregs as a mechanism to decrease airway inflammation associated with asthma. These findings expand our understanding of how androgens regulate type 2 immune responses, as previous findings showed that AR signaling decreased type 2 allergic airway inflammation by attenuating ILC2 proliferation and cytokine production and limiting Th2 cell differentiation and proliferation (41, 42, 44). Defining the mechanisms by which androgens and AR signaling reduce airway inflammation in asthma is critical for the personalization of therapeutics for men and women with asthma and the development of DHEA as a potential therapeutic for asthma. Further, results from this study may provide mechanisms for how AR signaling attenuates inflammation in other inflammatory diseases with a female predominance, including lupus and multiple sclerosis.

## Methods

### Animals.

BALB/c (BALB/cAnNCrl) mice were obtained from Charles River Laboratories. C57BL/6J (B6), BALB/c, C57BL/6J *Ar^Tfm^*, B6-*Foxp3^EGFP^* (B6.Cg-*Foxp3* C.Cg-*Foxp3^tm2Tch^*/J), BALB/c *Foxp3^EGFP^* (Cg-*Foxp3* C.Cg-*Foxp3^tm2Tch^*/J), and *DO11.10* [C.Cg-Tg(DO11.10)10Dlo/J] mice were purchased from The Jackson Laboratory and then bred in-house. *Ar^Tfm^* mice were crossed with B6-*Foxp3^EGFP^* mice to generate *Ar^Tfm^* *Foxp3^EGFP^* mice. *Foxp3*^EGFP-Cre^ *Rosa26^YFP/YFP^* *Il13^TdTomato^* mice were generated by crossing of *Foxp3*^EGFP-Cre^ *Rosa26^YFP/YFP^* and *Il13^TdTomato^* mice, gifts from Talal Chatila and Andrew McKenzie, respectively. *DO11.10* and *Foxp3^EGFP^* were crossed to generate *DO11.10 Foxp3^EGFP^* mice. *Il33^fl/fl^*eGFP mice were a gift from Paul Bryce (Northwestern University), and these mice are now commercially available at The Jackson Laboratory (stock 030619). *Il33^fl/fl^*eGFP (*Il33^EGFP^*) mice were crossed with *Ar^Tfm^* mice in-house to generate *Ar^Tfm^* *Il33^EGFP^* mice. *Ar^fl/fl^* and *Ar^fl/0^* mice were purchased from The Jackson Laboratory and bred with *Foxp3^YFP-Cre^* mice to generate *Ar^fl/0^* *Foxp3^Cre+^* mice. For all experiments, unless otherwise stated, mice 8–12 weeks old were used.

### Gonadectomy and administration of DHT or vehicle pellets.

In select experiments, at 3–4 weeks of age, male mice underwent a gonadectomy or a sham surgery. At 8 weeks of age, gonadectomized or sham-operated mice had 60-day slow-release pellets from Innovative Research of America containing DHT (15 mg) or vehicle subcutaneously implanted in the nape of the neck. Three weeks after implantation, *Alternaria alternata* extract (Alt Ext) challenge protocol was conducted as described below.

### Mouse model of allergic airway inflammation.

Mice were anesthetized with isoflurane and challenged intranasally with 5.0–7.5 μg (in 75 μL volume) of Alt Ext (lot 338869, Greer Laboratories) or vehicle (PBS) every 3 days for 4 total challenges as previously described or for less time as indicated (71). Twenty-four hours after the last challenge, mice were sacrificed for endpoint analysis. In select experiments, 300 ng of recombinant mouse IL-33 (rmIL-33; PeproTech) or vehicle (PBS) was administered intranasally to mice every 3 days for 4 challenges.

### DO11.10 Foxp3^EGFP^ iTreg adoptive transfer model.

As shown in Figure 2A, BALB/c recipient female mice were sensitized intraperitoneally at day 0 and day 7 with 100 μL solution of OVA (10 μg) conjugated to aluminum hydroxide (20 mg). On day 16, naive T cells were isolated from the spleens of *DO11.10* *Foxp3^EGFP^* male and female mice using a CD4^+^CD62L^+^ naive T cell isolation kit (catalog 130-106-643, Miltenyi Biotec) as previously described (67). To differentiated iTregs, naive T cells were plated at 100,000 cells per well on 24-well non–tissue culture flat-bottom plates precoated with 1 μg/mL anti-CD3 (catalog 553057, BD Biosciences) and supplemented with 100 IU/mL recombinant human IL-2 (NIH) and 10 ng/mL recombinant human TGF-β (PeproTech). On day 20, iTregs (live CD4^+^ *Foxp3*^EGFP+^ cells) were sorted by FACS. OVA-sensitized BALB/c recipient female mice were anesthetized with ketamine/xylazine, and 1.5 × 10^6^ sorted *DO11.10 Foxp3^EGFP^* iTregs from male or female mice were adoptively transferred retro-orbitally into BALB/c recipient female mice. On days 21–23, BALB/c recipient female mice were challenged with nebulized 1% OVA for 40 minutes each day. On day 24, BAL fluid and lungs were harvested from BALB/c recipient female mice, and OVA-specific iTregs were identified by flow cytometry using KJ126 antibody (Supplemental Table 1).

### BAL collection.

After euthanasia, BAL was performed by instillation of 800 μL of saline through a tracheostomy tube into the lungs and gentle withdrawal of fluid through the same 1 mL syringe. Cells from BAL were adhered to a slide and stained using a commercially available Three-Step Stain kit (Richard-Allan Scientific, Thermo Fisher Scientific). Eosinophils, neutrophils, lymphocytes, or monocytes were identified and categorized using light microscopy as previously described (40).

### Flow cytometry.

Lungs were harvested and digested using 1 mg/mL collagenase type IV (Sigma-Aldrich) and 0.02 mg/mL DNase I (Sigma-Aldrich) in RPMI with 10% FBS (Atlanta Biologics) for 30 minutes at 37°C as previously described (41, 72). In some experiments in which lungs were digested to obtain stromal cells, collagenase type I (Sigma-Aldrich) was used. To inactivate the enzymatic digestion, 1 μM EDTA was added, followed by filtering of the sample through a 70 μm strainer to remove debris. A red blood cell lysis was performed per the manufacturer’s instructions (BioLegend), and 2 million to 5 million viable cells were used for flow cytometry assays. Cells were stained with a fixable viability dye (Ghost Dye UV450, Tonbo Biosciences) and blocked using an anti–mouse FcR (CD16 and CD32) antibody. Cells were then stained for surface antigens (Supplemental Table 1) for 45 minutes followed by fixation and permeabilization. After fixation and permeabilization, cells were stained for intracellular markers as shown in Supplemental Table 1. All flow cytometry was conducted on a BD LSR II flow cytometer (BD Biosciences) or a Cytek Aurora (Cytek Biosciences), and the data were analyzed using FlowJo (BD Biosciences).

For the iTreg adoptive transfer experiments, the single-cell suspension was stimulated for 4 hours at 37°C using 1 μM ionomycin (Sigma-Aldrich), 50 ng/mL PMA (Sigma-Aldrich), and 0.07% GolgiStop (BD Biosciences). Cells were then counted and processed for cell surface and intracellular staining as mentioned above.

### Treg suppression assay.

CD4^+^ T cells were enriched from the spleens of B6-*Foxp3^EGFP^* male, B6-*Foxp3^EGFP^* female, and *Ar^Tfm^* *Foxp3^EGFP^* male mice using a CD4^+^ T cell isolation kit (catalog 130-104-454, Miltenyi Biotec). Splenic Tregs (live CD4^+^ *Foxp3^EGFP+^*) were isolated by FACS from B6-*Foxp3^EGFP^* male and female and/or *Ar^Tfm^* *Foxp3^EGFP^* male mice. At the same time, CD4^+^ T effector cells (live CD4^+^ Foxp3*^EGFP–^*) were isolated by FACS from B6-*Foxp3^EGFP^* female mice. Total splenocytes were also isolated from C57BL/6 female mice and irradiated using 35 Gy of cesium-137 γ-radiation to serve as feeder cells. T effectors were incubated for 20 minutes with CellTrace Violet dye (catalog C34571, Thermo Fisher Scientific) per the manufacturer’s directions. Tregs and T effector cells were cultured at Treg/T effector ratios of 0:1, 1:1, 1:2, 1:4, and 1:8 in the presence of irradiated splenocytes using T cell medium (RPMI 1640 Medium with l-glutamine [11875-093, Gibco] + 10% FBS [50 mL] + 1% penicillin/streptomycin [5 mL] + 1% HEPES [Gibco; 5 mL of 1 M solution] + 1% Na pyruvate [Gibco; 5 mL of 100 mM] + 1.8 μL β-mercaptoethanol [Sigma-Aldrich; final concentration 55 μM]) and stimulated with 1 μg/mL anti-CD3 (catalog 100340, Ultra-LEAF Purified anti–mouse CD3ε antibody, BioLegend) in a tissue culture–treated 96-well plate. Cells remained in culture for 4 days, and then flow cytometry was conducted to determine T effector cell proliferation by measurement of CellTrace Violet (Thermo Fisher Scientific). Proliferation of T effector cells in the absence of Tregs (Treg/T effector ratio 0:1) was considered 100%. Percent Treg suppression was determined as 1 minus percent of proliferating T effectors times 100 for each Treg/T effector ratio.

### ELISA.

Following the manufacturer’s instructions, cytokine levels were measured by Duoset and Quantikine ELISA kits (R&D Systems). Any value below the lower limit of detection was assigned half the value of the lowest detectable standard to conduct statistical analysis.

### Airway hyperresponsiveness.

Mice were anesthetized with pentobarbital sodium (85 mg/kg), and an 18-gauge tracheostomy tube was placed in the trachea of the mice. Mice were then mechanically ventilated using a Sci-Req FlexiVent machine with 150 breaths/min and a tidal volume of 2 mL. Airway resistance was determined at baseline and after administration of increasing doses of nebulized acetyl-β-methacholine (0–100 mg/mL; Sigma-Aldrich).

### Histopathology.

Forty-eight hours after the last Alt Ext or PBS challenge, lungs were perfused and fixed in 10% formalin at 4°C for 24 hours. Lungs were then embedded in paraffin blocks, and tissue sections (5 μm) were stained with H&E to assess inflammation or periodic acid–Schiff (PAS) to assess goblet cell hyperplasia as a measure of mucus production. Slides were scored by a pathologist who was blinded to the experimental groups. For inflammation (H&E staining), a 0–3 scoring system was used: 0, no inflammatory cells; 1, few inflammatory cells; 2, increased accumulation of inflammatory cells; 3, abundant accumulation of inflammatory cells. For mucus production, individual airways were scored for goblet cell metaplasia according to the following scale: 0, no PAS-positive cells; 1, <5% PAS-positive cells; 2, 5%–10% PAS-positive cells; 3, 10%–25% PAS-positive cells; 4, >25% PAS-positive cells.

### Quantitative PCR.

Total RNA was extracted using the RNeasy Mini Kit (Qiagen). cDNA was normalized to 200 ng of total RNA and synthesized using SuperScript IV First-Strand Synthesis System (Thermo Fisher Scientific). Gene expression was determined using TaqMan primers purchased from Applied Biosystems, and relative expression was normalized to the housekeeping gene *Gapdh* as previously described (41).

### Microarray analysis on GEOquery data.

Microarray analysis was performed using the R statistical computing program (version 4.1.0). The R library GEOquery (version 2.60.0; https://www.ncbi.nlm.nih.gov/geo/) was used to download the normalized microarray data for 4 different asthma studies (GSE4302, GSE63142, GSE43696, GSE41861). Probe sets mapping to a given gene symbol were averaged across each sample. The sex variable was not available for studies GSE4302 and GSE63142 and was imputed based on clustering genes on the Y chromosome. Briefly, the expression values for the genes on the Y chromosome were extracted, and principal component analysis was applied. Using the first 2 principal components, samples were classified into 2 clusters based on *k*-means clustering. Samples with expression of genes on the Y chromosomes were labeled as males. For each study, the expression of *AR* was compared between males and females using a Student’s *t* test.

### hBE33 cells and Alt Ext exposure.

hBE33 cells are an immortalized human airway epithelial cell line derived from a male donor and engineered to stably express IL-33eGFP (73). hBE33 cells are maintained in BronchiaLife medium supplemented with grown factors and retinoic acid (Lifeline Cell Technology) as well as 1% penicillin/streptomycin. Once confluent, hBE33 cells were pretreated with 5α-DHT for 24 hours prior to exposure to Alt Ext (30 μg/mL) for 1 hour, and then basolateral supernatants were collected.

### Primary HBE cell culture and Alt Ext exposure.

Primary HBE cells were obtained as previously described (74–76). Cells were cultured on 0.03 mg/mL type I rat collagen–coated Transwells (Costar) using PneumaCult Ex-Plus Medium (StemCell Technologies) plus supplements including hydrocortisone (StemCell Technologies), penicillin, and streptomycin (Gibco). Once a monolayer was established, HBE cells were differentiated at the air-liquid interface with PneumaCult Ex-Plus Medium plus supplements in the basolateral chamber for 14 additional days. Differentiated HBE cells were pretreated with 5α-DHT in the basolateral chamber for 24 hours prior to exposure to Alt Ext (30 μg/mL) at the apical surface for 1 hour.

### Human lung samples and flow cytometry.

CD45^+^ cells were isolated from fresh excised human lung tissue obtained from deceased organ donors whose lungs were determined not to be suitable for transplantation, using a Miltenyi isolation kit (130-045-801), and were cryopreserved in 10% DMSO (Sigma-Aldrich) and FBS. Cells were quickly thawed and activated with anti–human CD3/CD28/CD2 antibody bead solution (Miltenyi Biotec) per the manufacturer’s directions in the presence of 200 U human IL-2, 40 ng/mL recombinant human IL-33, 5α-DHT (1 nM), and/or vehicle (methanol). Twenty-four hours later, cells were collected and stained with viability dye (Live/Dead Aqua, Thermo Fisher Scientific) followed by surface staining with redFluor–anti-CD45, Pacific blue–anti-CD3, FITC–anti-CD4, APC-Cy7–anti-CD25, and APC–anti-ST2 antibodies as previously described (76). Cells were then permeabilized and stained with PECF594–anti-GATA3 and PE-Cy7–anti-Foxp3 as previously described (76). Flow cytometry was conducted on a BD LSR II flow cytometer, and the data were analyzed using FlowJo (BD Biosciences).

### IL-33 stimulation of splenic Tregs.

Splenic Tregs were isolated as described above for Treg suppression assay. Tregs were activated with 1 μg/mL anti–mouse CD3 and 0.5 μg/mL anti–mouse CD28 antibodies in the presence of recombinant human IL-2 (100 IU). Cells were also treated with 0.1 to 1 nM 5α-DHT, methanol (vehicle for 5α-DHT), 100 ng/mL IL-33, and/or PBS (vehicle for IL-33) depending on the experimental group. Tregs were activated for 3 days, and then cell pellets were harvested for RNA extraction and quantitative PCR as described above.

### Statistics.

All statistical analysis was performed using GraphPad Prism 9. Data are represented as mean ± SEM. Comparisons between 2 groups were made using a 2-tailed Student’s *t* test or Mann-Whitney test for parametric or nonparametric data, respectively. Comparisons between more than 2 groups were made using 1-way ANOVA followed by Tukey’s post hoc test or ANOVA for repeated measures followed by Tukey’s post hoc test as indicated for each experiment. Data were considered significant when *P* was less than 0.05.

### Study approval.

All human tissue and cells used in this study were deidentified and IRB exempt. All breeding, surgical procedures, and animal experiments were approved by the Institutional Animal Care and Use Committee at Vanderbilt University Medical Center and conducted according to the *Guide for the Care and Use of Laboratory Animals* (National Academies Press, 2011).

## Author contributions

VDG, JYC, and DCN designed the studies, performed experiments, analyzed data, and wrote the manuscript. NUC and ET designed studies, performed experiments, and analyzed data. AEN and RSP provided expertise and mice as well as reviewed the manuscript. JZ, ZC, JYC, NUC, and AEN maintained mouse colonies and genotyping for studies. VVP is a pathologist who scored the histology slides and conducted microscopy. AS provided bioinformatics support on GEOqueries. LBW, JAB, CMS, NDP, and NW provided CD45^+^ cells from excised lungs. HWC provided the HBE cells and expertise on expanding and differentiating cells. Order of first authors was determined by VDG initiating the project and providing approximately half of the data included in this article. JYC finished the remaining half of the project, warranting a coauthorship.

## Supplementary Material

Supplemental data

## Version 1 01/13/2022

In-Press Preview

## Version 2 02/15/2022

Electronic publication
