# Supplementary material for: Androgen receptor signaling promotes Treg suppressive function during allergic airway inflammation
Source: J Clin Invest. 2022 Feb 15;132(4):e153397. doi: 10.1172/JCI153397 (PMC8843736; doi:10.1172/JCI153397)
Supplement: Supplemental data [file jci-132-153397-s191.pdf]

Androgen receptor signaling promotes Treg suppressive function during allergic airway inflammation

Vivek D. Gandhi[1*], Jacqueline-Yvonne Cephus[1*], Allison E. Norlander[1], Nowrin U. Chowdhury[2], Jian Zhang[1], Zachary Ceneviva[1], Elie Tannous[1], Vasiliy V Polosukhin[1], Nathan D. Putz[1], Nancy Wickersham[1], Amrit Singh[3], Lorraine B. Ware[1], Julie A. Bastarache[1], Ciara M. Shaver[1], Hong Wei Chu[4], R. Stokes Peebles, Jr.[1,2], Dawn C. Newcomb[1,2]

[1] Department of Medicine, Vanderbilt University Medical Center, Nashville, TN, USA

[2] Department of Pathology, Microbiology, and Immunology, Vanderbilt University Medical Center, Nashville, TN, USA

[3] Prevention of Organ Failure (PROOF) Centre of Excellence, University of British Columbia, Vancouver, BC, Canada

[4]National Jewish Medical Center, Denver, CO, USA

* contributed equally to this work.

Corresponding author:

Dawn C. Newcomb, PhD

T-1218 Medical Center North

1161 21st Avenue South

Nashville, TN 37232

(615) 875-7782

dawn.newcomb@vumc.org

Conflicts of interest: The authors have declared that no conflict of interest exists.

Supplement Table – Antibodies, viability dyes, and isolation kits used in this project

Reagent	Antibody target	Clone	Fluorophore	Manufacturer	Catalog Number
anti-mouse antibody	Bcl6	K112-91	Alexa Fluor 647	BD Biosciences	561525
anti-mouse antibody	CD16/CD32	2.4G2	Purified	BD Biosciences	553142
anti-mouse antibody	CD25	PC61	APC-Cy7	BD Biosciences	557658
anti-mouse antibody	CD28	37.51	Ultra-LEAF purified	BioLegend	102116
anti-mouse antibody	CD3	17A2	Alexa Fluor 700	BioLegend	100216
anti-mouse antibody	CD3	145-2C11	APC-Cy7	BD Biosciences	557596
anti-mouse antibody	CD3e	145-2C11	Ultra-LEAF purified	BioLegend	100340
anti-mouse antibody	CD4	GK1.5	Alexa Fluor 700	BioLegend	100429
anti-mouse antibody	CD4	GK1.5	FITC	eBioscience	11-0041-82
anti-mouse antibody	CD4	H129.19	PE-Cy5	BD Biosciences	553654
anti-mouse antibody	CD4	RM4-5	PE-Cy7	BioLegend	100527
anti-mouse antibody	CD4	RM4-5	PerCP-Cy5.5	BD Biosciences	550954
anti-mouse antibody	CD90	53-2.1	Brilliant Violet 786	BD Biosciences	564365
anti-mouse antibody	CTLA4	UC10-4F10-11	APC	BD Biosciences	564331
anti-mouse antibody	DO11.10 TCR	KJ126	Biotin	eBioscience	13-5808-82
anti-mouse antibody	ICOS	C398.4A	PE-Cy7	eBioscience	25-9949-82
anti-mouse antibody	IL-13	eBio13A	PerCP-Cy5.5	eBioscience	46-7133-80
anti-mouse antibody	LAG-3	C9B7W	Brilliant Violet 786	BD Biosciences	740959
anti-mouse antibody	ST2	U29-93	Brilliant Violet 421	BD Biosciences	566309
anti-mouse antibody	ST2	U29-93	PE	BD Biosciences	566311
anti-mouse antibody	Streptavidin		PECF594	BD Biosciences	562284
anti-human antibody	CD2, CD3, and CD28		T Cell Activation/Expansion Kit, human	Miltenyi Biotec	130-091-441
anti-human antibody	CD25	BC96	APC-Cy7	BioLegend	302614
anti-human antibody	CD3	UCHT1	PacBlue	BD Biosciences	558117
anti-human antibody	CD4	SK3	FITC	BD Biosciences	562970
anti-human antibody	CD45	HI30	redFluor 710 (AF700)	Tonbo Biosciences	80-0459
anti-human antibody	ST2		APC	R&D Systems	FAB5231A
anti-mouse/human antibody	Foxp3	FJK-16S	PE-Cy7	eBioscience	25-5773-82
anti-mouse/human antibody	GATA3	L50-823	PECF594 (TxREd)	BD Biosciences	563510
Cell viability dye			Live/Dead Aqua	ThermoFisher	L34957
Cell viability dye			Ghost Dye UV 450	Tonbo Biosciences	13-0868
Cell viability dye			Ghost Dye Violet 510	Tonbo Biosciences	13-0870
Cell proliferation dye			Cell Trace Violet	ThermoFisher	C34571
CD4+ CD62L+ naïve T cell isolation kit				Miltenyi Biotec	130-106-643
CD4+ T cell isolation kit				Miltenyi Biotec	130-104-454

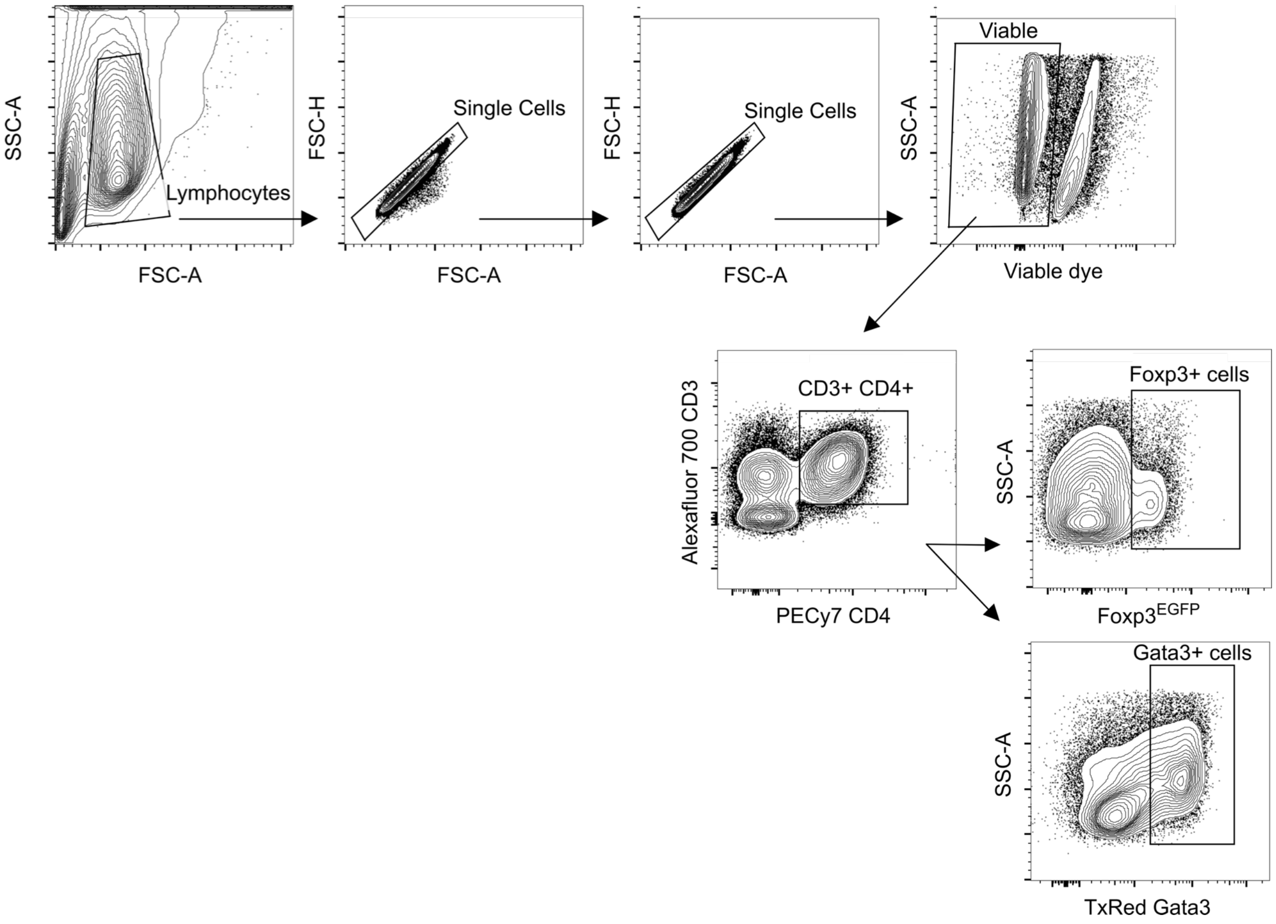

Supplemental Figure 1. Gating strategy for identification of Tregs (CD3+ CD4+ Foxp3+) and Th2 cells (CD3+ CD4+ GATA3+). Representative sample showing gating strategy.

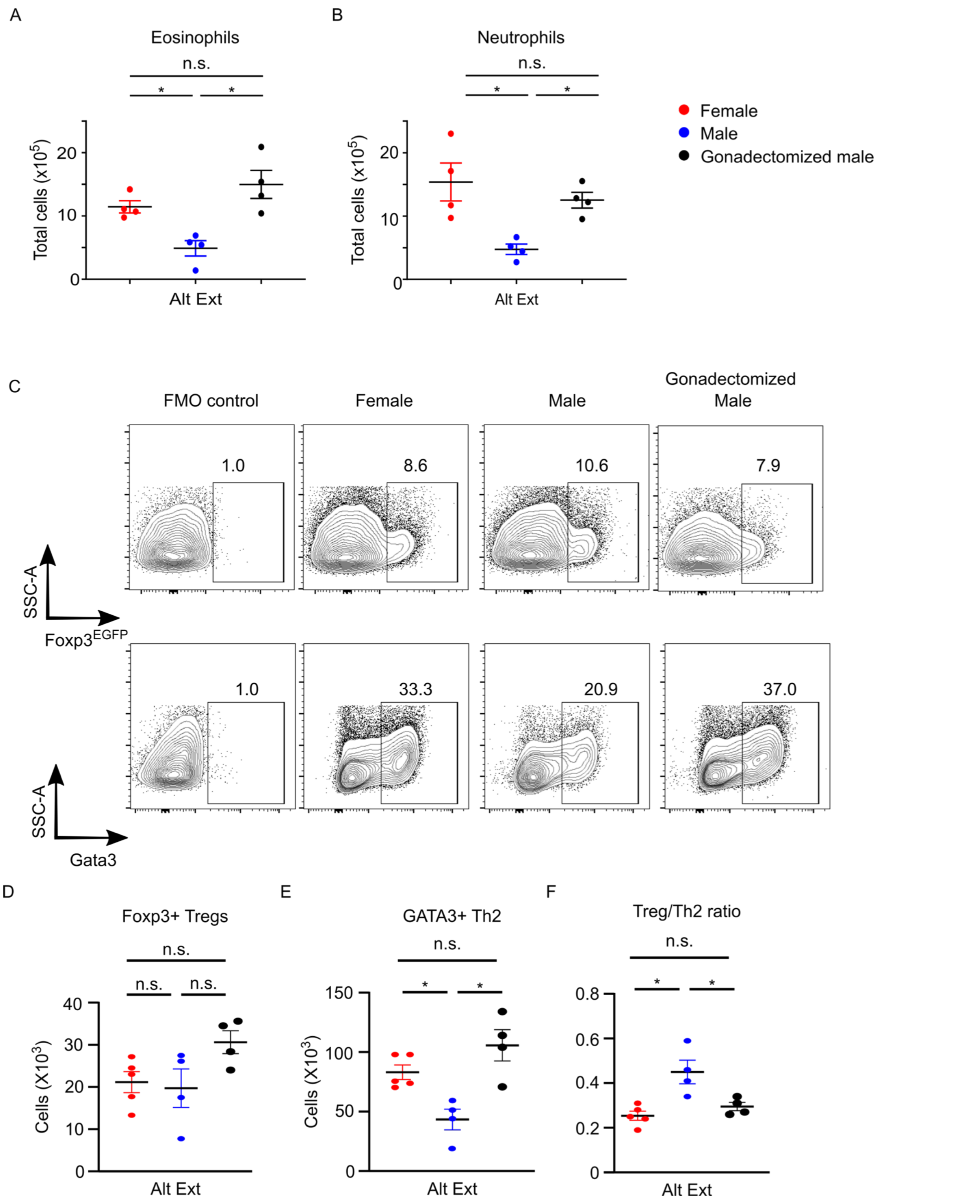

Supplemental Figure 2. Testosterone decreases Alt Ext induced allergic inflammation and increases Treg to Th2 cell ratio. Sham-operated C57BL/6J male and female mice and gonadectomized male mice underwent the Alt Ext protocol. BAL fluid and lungs were harvested 1 day following the last challenge. **(A-B).** Eosinophils and neutrophils in BAL fluid. **(C)** Representative dots plots showing gating for Tregs and Th2 cells in lung (cells were pre-gated on viable, CD3+ CD4+ cells). **(D-F).** Quantification of Tregs, Th2, and the Treg to Th2 ratio. Data are mean ± SEM; n = 4-5, * $p<0.05$, ANOVA with Tukey post-hoc.

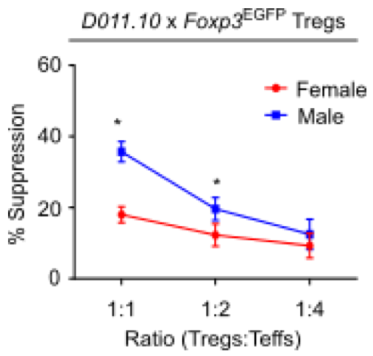

Supplemental Figure 3. Male Tregs are more suppressive than female Tregs in *D011.10 x Foxp3*^{EGFP} mice. Treg suppression assay using Tregs from *D011.10 x Foxp3*^{EGFP} female or male mice and T effector cells (Teffs) from *D011.10* female mice. Percent suppression of Tregs at various ratios of Tregs to Teffs. Data are mean ± SEM; n = 3, representative data from 2 experiments * $p<0.05$, ANOVA with Tukey post-hoc.

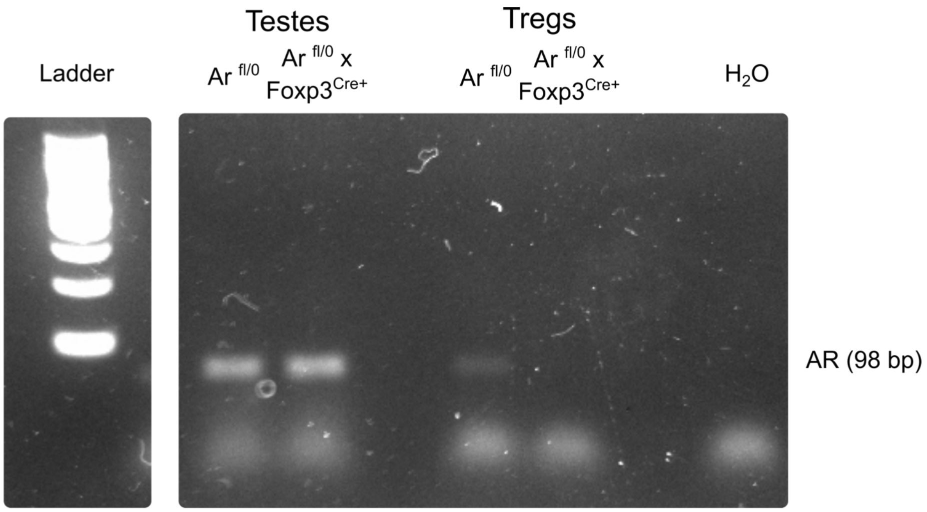

Supplemental figure 4. PCR verification of AR deficiency in Tregs. PCR was conducted on testes and splenic Tregs from *Ar*$^{fl/0}$ and *Ar*$^{fl/0}$ x *Foxp3*$^{Cre+}$ male mice. Water was used as a negative control.

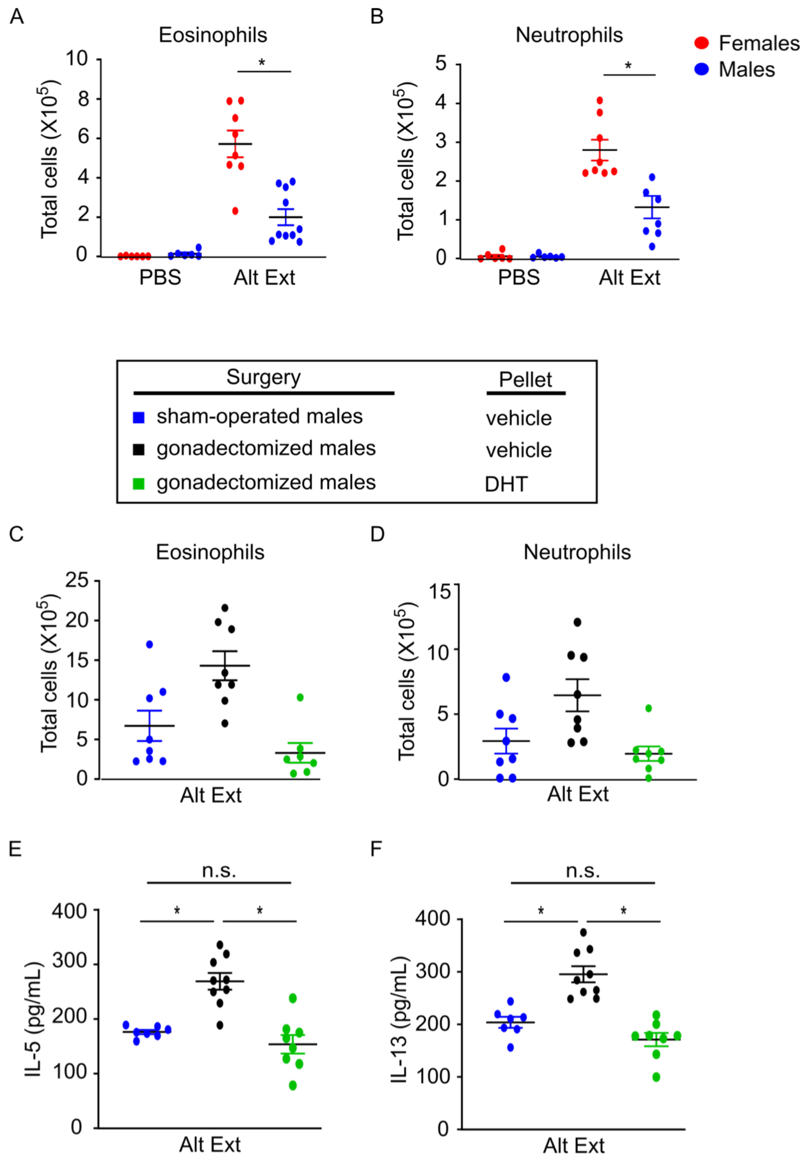

Supplemental figure 5. AR signaling decreases Alt Ext-induced airway inflammation in *Foxp3*$^{\mathbf{GFP/YFP}}$ x *Il13*$^{\mathbf{Tdtomato}}$ mice. (A-B) BAL eosinophils and neutrophils in female and male *Foxp3*$^{GFP/YFP}$ x *Il13*^{Tdtomato} mice. **(C-F)** *Foxp3*$^{GFP/YFP}$ x *Il13*^{Tdtomato} male mice underwent gonadectomy or sham operation at 3-4 weeks of age. At 8 weeks old, 5α-DHT (DHT) or vehicle slow-release pellets were subcutaneously placed into mice. After 3 weeks, mice underwent Alt Ext protocol. **(C-D)** BAL eosinophils and neutrophils. **(E-F)** IL-5 and IL-13 levels in whole lung homogenates. Data are mean ± SEM; n = 6-10 from 2 separate experiments, * $p<0.05$, ANOVA with Tukey post-hoc.

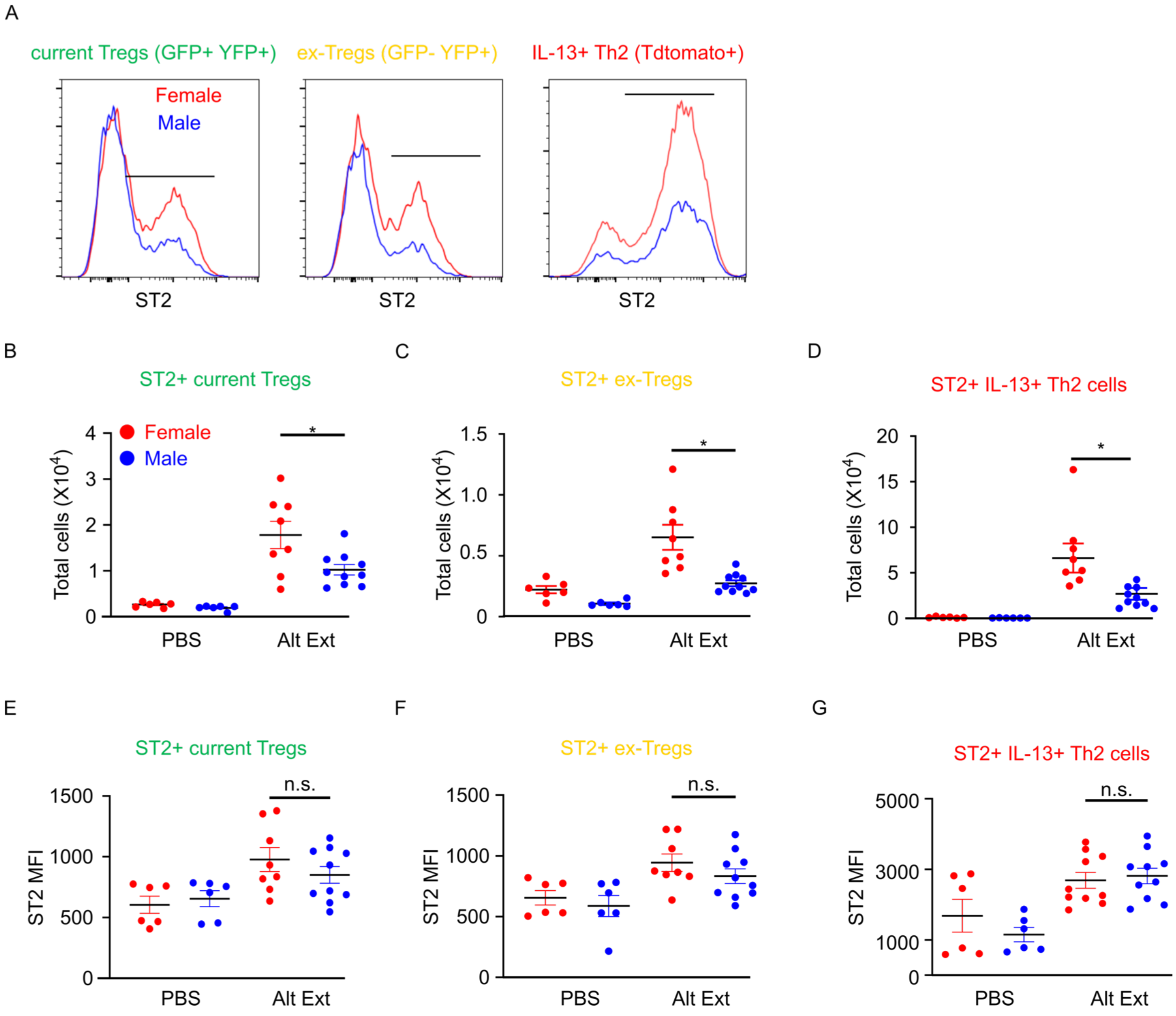

Supplemental figure 6. Males have decreased numbers of ST2+ Tregs after Alt Ext challenge. (A) Representative histograms of ST2+ current Tregs, ex-Tregs, or Th2 cells from *Foxp3*$^{GFP/YFP}$ x *Il13*^{Tdtomato} female and male mice. **(B-D)** Numbers of ST2+ current Tregs, ex-Tregs, and IL-13+ Th2 cells in lungs of mice. **(E-G)** ST2 MFI. Data are mean ± SEM; n = 6-10 from 2 separate experiments, * p<0.05, ANOVA with Tukey post-hoc.

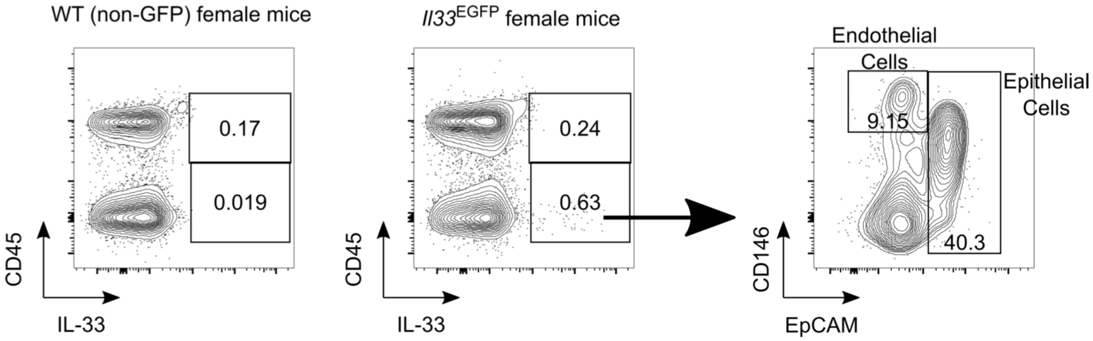

Supplemental Figure 7. Gating strategy for identification *IL-33*$^{\mathbf{EGFP}}$+ cells.
Representative dot plots showing that epithelial cells were identified as CD45- EpCAM+, endothelial cells were identified as CD45- EpCAM- CD146+, and immune cells were identified as CD45+ cells.

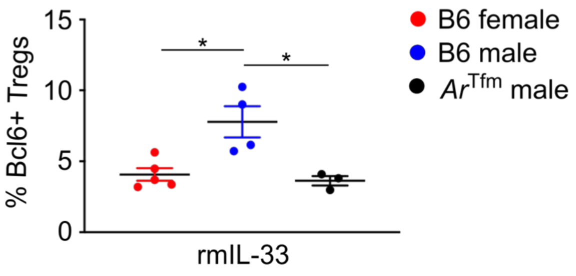

Supplemental Figure 8. AR signaling increases Bcl6+ Tregs. Bcl6+ Tregs were determined from rmIL-33 challenged female, male, and *Ar*^{Tfm} male mice. Data are mean ± SEM; n = 3-5, * $p<0.05$, ANOVA with Tukey post-hoc.
